# Human pancreatic cancer stem cells are sensitive to dual inhibition of IGF-IR and ErbB receptors

**DOI:** 10.1186/s12885-015-1249-2

**Published:** 2015-04-04

**Authors:** Nerea Urtasun, Anna Vidal-Pla, Sandra Pérez-Torras, Adela Mazo

**Affiliations:** 1 Departament de Bioquímica i Biologia Molecular, Universitat de Barcelona, Barcelona, Spain; 2 Institut de Biomedicina de la Universitat de Barcelona (IBUB), Barcelona, Spain; 3 CIBERehd, Madrid, Spain

**Keywords:** Pancreatic ductal adenocarcinoma, Cancer stem cells, IGF-IR, EGFR, Her-2

## Abstract

**Background:**

Pancreatic ductal adenocarcinoma is a particularly challenging malignancy characterized by poor responsiveness to conventional chemotherapy. Although this tumor frequently overexpresses or possesses constitutively activated variants of IGF-IR and EGFR/Her-2, clinical trials using inhibitors of these receptors have failed. ErbB receptors have been proposed as one mechanism involved in the resistance to IGF-IR inhibitors. Therefore, combined treatment with inhibitors of both IGF-IR and ErbB receptors would appear to be a good strategy for overcoming the emergence of resistance.

**Methods:**

Sensitivity of cells to NVP-AEW541 and lapatinib in single or combination treatment was assessed by MTT or WST-8 assays in a panel of human pancreatic cancer cell lines and cancer stem cells. Tumorspheres enriched in cancer stem cells were obtained from cultures growing in non-adherent cell plates. The effects on cell signalling pathways were analyzed by Western blot.

**Results:**

We found that combined treatment with the IGF-IR and EGFR/Her-2 inhibitors NVP-AEW541 and lapatinib, respectively, synergistically inhibited pancreatic cancer cell growth. Analysis at molecular level argued in favor of cross-talk between IGF-IR and ErbBs pathways at IRS-1 level and indicated that the synergistic effect is associated with the total abolishment of Akt, Erk and IRS-1 phosphorylation. Moreover, these inhibitors acted synergistically in tumorsphere cultures to eliminate cancer stem cells, in contrast to their resistance to gemcitabine.

**Conclusions:**

Taken together, these data indicate that simultaneous blockade of IGF-IR and EGFR/Her-2 using NVP-AEW541 and lapatinib may overcome resistance in pancreatic cancer. Thus, the synergy observed with this combined treatment indicates that it may be possible to maximize patient benefit with the appropriate combination of currently known anticancer agents.

**Electronic supplementary material:**

The online version of this article (doi:10.1186/s12885-015-1249-2) contains supplementary material, which is available to authorized users.

## Background

Pancreatic ductal adenocarcinoma (PDAC) is one of the five most common causes of cancer death, owing to its late diagnosis, high dissemination at early stages, and poor responsiveness to both radio- and chemotherapy [1]. Gemcitabine remains the current standard first-line treatment [2]. However, chemotherapy in advanced disease confers only modest survival advantage and symptoms palliation. Recent clinical trials of gemcitabine combination therapies have produced significant, but low, response rates in advanced pancreatic cancer, underscoring the need for new therapeutic approaches [3-5].

An important consideration in these strategies is the heterogeneity of pancreatic tumors. In this context, several studies investigating pancreatic cancer biology have identified a subpopulation of cells termed pancreatic cancer stem cells (PCSCs) [6-8]. This subpopulation may play a critical role in the resistance to chemotherapy and radiation, suggesting that such cells may be the source of some cases of pancreatic cancer relapse [9,10]. Therefore, therapeutic modalities that lead to the elimination of CSCs could improve clinical outcome in patients with pancreatic cancer.

Receptor tyrosine kinases are currently among the most promising therapeutic targets in a wide range of tumors. Inhibition of receptor tyrosine kinases of the ErbB family has been approved for the treatment of different tumors and is used extensively to treat breast cancer [11]. There has also been growing research interest in insulin-like growth factor-1 receptor (IGF-IR) as a target for antitumor therapy [12-14], given the demonstrated ability of IGF-IR to potently contribute to a variety of oncogenic effects, including cell proliferation, cell survival, and cell differentiation [15-17]. IGF-IR is frequently overexpressed or activated in pancreatic cancer, a factor that most likely contributes to the aggressive growth characteristics and poor prognosis of these tumors [18-20]. Moreover, molecular mechanisms that lead to autocrine activation of the IGF-IR and stimulation of downstream signaling through phosphorylation (activation) of Akt have been identified and could further substantially contribute to tumor progression and invasion [21,22].

On the basis of these findings, IGF-IR has come to be viewed as a rational therapeutic target in pancreatic cancer, prompting clinical investigations of IGF-IR inhibitors. However, recent clinical trials of anti-IGF-IR compounds in combination with gemcitabine have failed to demonstrate improved patient survival [23,24], a failure attributable, at least in part, to the development of resistance. One mechanism proposed to account for resistance is activation of alternative survival pathways [25]. Among these candidate alternative pathways are those activated by members of the ErbB receptor family, which are important in regulating cell survival [26-28] and are frequently overexpressed in pancreatic carcinomas [29,30]. Importantly, activation of mitogen-activated protein kinases (MAPKs) by ErbB receptors signaling may counterbalance the decrease in phosphorylated Akt induced by IGF-IR inhibitors. This compensatory mechanism could explain the failure of treatments based on individual inhibition of IGF-IR or ErbB [14,28,31], and suggests that therapeutic strategies based on combined inhibition of IGF-IR and ErbB receptors could overcome this resistance.

In the current study, we tested this hypothesis, investigating the impact of concurrent inhibition of IGF-IRs and epidermal growth factor receptors (EGFR/Her-2) by NVP-AEW541 and lapatinib tyrosine kinase inhibitors, respectively, on pancreatic cancer cell lines and particularly on PCSCs.

## Methods

### Reagents and immunochemicals

Lapatinib was kindly provided by GlaxoSmithKline (Brentford, UK) and NVP-AEW541 was a kind gift of Novartis Pharma (Basel, Switzerland). Stock solutions of drugs were prepared in dimethyl sulfoxide and stored at −20°C, and diluted in fresh media before each experiment. Insulin-like growth factor (IGF-I) and Epidermal growth factor (EGF) (Peprotech, Rocky Hill, NJ, USA) were dissolved in phosphate-buffered saline containing 0.1% bovine serum albumin (BSA). Cells were immunostained using antibodies against EGFR (1005), Her-2 (C-18), Her-3 (C-17), IGF-IRβ (C-20) and Akt-1 (C-20) (Santa Cruz Biotechnology, Santa Cruz, CA, USA); phospho-Akt (Ser473), phospho-p44/42 (Thr202/Tyr204) and p44/42 (137 F5) (Cell Signaling Technology, Danvers, MA, USA); phospho-IRS-1 (Tyr612) and phospho-IRS-1 (Tyr896) (Invitrogen, Camarillo, CA, USA); and β-actin (Sigma-Aldrich, St. Louis, MO, USA).

### Cell culture

NP-9, NP-18, and NP-29 cell lines (kindly provided by Dr Capella from Hospital de la Santa Creu i Sant Pau, Barcelona, Spain) were derived from human pancreatic adenocarcinomas xenografted in nude mice [32]. The BxPC3 cell line was obtained from the American Type Culture Collection (Manassas, VA, USA). CP15T and CP15A cell lines were also derived from a human pancreatic adenocarcinoma xenografted in nude mice by our group [33]. The research protocol complied with the ethical guidelines of the 1975 Declaration of Helsinki and was approved by the ethics committee of Universitat de Barcelona. All participants provided written informed consent. NP-9, NP-29, CP15T and CP15A cells were grown in a 1:1 mixture of Dulbecco’s modified Eagle’s medium (DMEM) and F12 medium; BxPC3 cells were grown in DMEM; and NP-18 cell were grown in RPMI-1640 medium (Gibco, Grand Island, NY, USA). All media were supplemented with 5% fetal bovine serum and antibiotics (penicillin/streptomycin). Cells were maintained in a humidified atmosphere of 5% CO_2_ at 37°C and subcultured every 3–4 days.

For tumorsphere cultures, cells were grown in ultra-low attachment plates (Corning, Gendale, AZ, USA) using serum-free DMEM:F12 (1:1) supplemented with B-27, N2, antibiotic-antimycotic (Invitrogen), 20 ng/ml human EGF, and 20 ng/ml human basic fibroblast growth factor (bFGF; Peprotech). Tumorspheres were dissociated weekly using trypsin and maintained for several passages. Experiments were performed between the fourth and seventh passage [34].

### Dose–response assays

Dose–response assays were performed by seeding 2–5 × 10^3^ cells/well in 96-well culture plates. Cultures were exposed to increasing concentrations of lapatinib and/or NVP-AEW541 for 72 h, at which time cell viability was determined by MTT (3-[4,5-dimethylthiazol-2-yl]-2,5 diphenyl tetrazolium bromide) assay.

Assays comparing monolayers and tumorspheres were performed by seeding single-cell suspensions at a density of 1.5 × 10^3^ cells/well in standard or ultra-low-adhesion 96-well culture plates, respectively, with increasing concentrations of lapatinib and/or NVP-AEW541. Cell viability was determined 72 h post-treatment using a WST-8 assay (Sigma-Aldrich), as described by the manufacturer.

Data were fitted to a dose–response curve using standard nonlinear regression, adapting a Hill equation with Grafit software (Erithacus Software, Ltd., Horley, UK) to obtain 50% inhibitory concentration (IC_50_) values. Cell survival for all experiments is expressed as the percentage of viable cells relative to that in untreated cells (defined as 100%).

The coefficient of drug interaction (CDI) was used to analyze the effect of drug combination. CDI was calculated based on the absorbance in each group, as CDI = AB/(A × B), where AB is the ratio for the combination group relative to the control group, and A and B are the ratios of each single agent group relative to the control group. Thus, a CDI value < 1 indicates synergy, a CDI value = 1 indicates additive effects, and a CDI value > 1 indicates antagonism. CDIs less than 0.7 indicate a significant synergistic effect.

### Protein extraction and Western blot

Cells were lysed in ice-cold lysis buffer containing 20 mM Tris (pH 8), 150 mM NaCl, 10 mM EDTA, 10 mM Na_4_P_2_O_7_, 2 mM VO_4_^3−^, 100 mM NaF, 1 mM β-glycerophosphate, 1% NP40, and protease and phosphatase inhibitor cocktails (Roche Applied Sciences, Penzberg, Germany). Lysates containing equal amounts of protein (20 μg for monolayer experiments and 30 μg for experiments comparing monolayers and tumorspheres), assessed by Bradford assay (Bio-Rad, Hercules, CA, USA), were electrophoretically separated on 8% polyacrylamide-sodium dodecyl sulfate gels and transferred to nitrocellulose membranes (Schleicher and Schuell, Dassel, Germany). Membranes were immunoblotted with the indicated primary antibodies. Antibody labeling was detected using an enhanced chemiluminescence detection kit (Biological Industries, Kibbutz Beit Haemek, Israel).

## Results

### Sensitivity of human pancreatic cancer cell lines to NVP-AEW541 and lapatinib

Expression levels of IGF-IR and ErbB family receptors were examined in a panel of human pancreatic cancer cell lines. IGF-IR expression levels varied, with high levels detected in NP-29 and CP15A cell lines. Notably, the highest levels of EGFR expression were also found in NP-29 cells, whereas EGFR expression was negligible in CP15T and CP15A cells. In contrast, Her-2, which was observed in all cell lines, showed marked expression in CP15T and CP15A cells. Her-3 expression was only clearly detectable in NP-29, CP15T, and CP15A cells.

**Figure 1:**
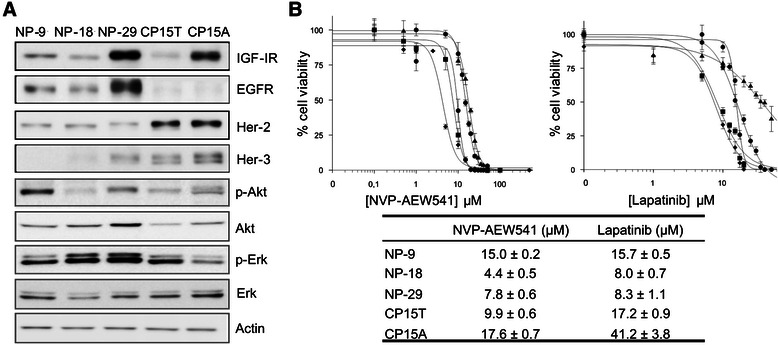
**Inhibition of IGF-I and ErbB receptors with NVP-AEW541 and lapatinib in pancreatic cancer cell lines. (A)** Basal levels of IGF-I and ErbB receptors and their signaling pathway components. Cells cultured to approximately 90% confluence were lysed and proteins in lysates were analyzed by Western blot. **(B)** Dose–response curves and IC_50_ values for NVP-AEW541 and lapatinib in the panel of cell lines. Cells were treated 24 h after seeding with increasing concentrations of NVP-AEW541 or lapatinib, and cell viability was measured by MTT assay 72 h after the start of treatment. Data are presented as means ± standard deviation of a representative experiment (n = 3). ● NP-9, ♦ NP-18, ■ NP-29, CP15T, ▲ CP15A.

Intracellular signaling pathways were assessed by evaluating Akt and Erk (extracellular signal-regulated kinase) phosphorylation. These experiments revealed a range of activation levels, with NP-9 cells showing the highest levels of Akt phosphorylation and CP15A cells showing the lowest levels of Erk phosphorylation (Figure 1A).

The effects of the IGF-IR inhibitor, NVP-AEW541, and the EGFR and Her-2 inhibitor, lapatinib were then examined in all five cell lines. NVP-AEW541 induced a concentration-dependent inhibition of growth in all cell lines. IC_50_ values ranged from 4.4 to 17.6 μM, with the most potent effect observed in NP-18 cells. Lapatinib also induced concentration-dependent growth inhibition in all cell lines. Again, NP-18 cells showed the highest sensitivity, and IC_50_ values ranged from 8.0 to 41.2 μM (Figure 1B).

### Response of pancreatic cancer cells to combined IGF-IR and EGFR/Her-2 inhibition

**Figure 2:**
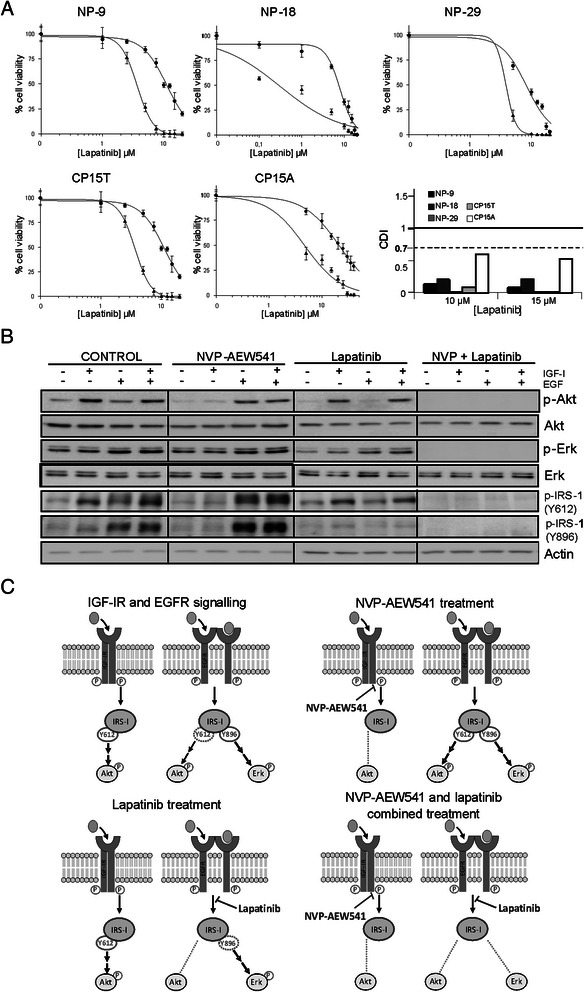
**Effect of NVP-AEW541 and lapatinib combined treatment on the growth of pancreatic cancer cell lines. (A)** Dose–response curves and CDI values for NVP-AEW541 and lapatinib combinations. Twenty-four hours after seeding, cells were treated with increasing concentrations of lapatinib alone (●) or combined with a fixed concentration of NVP-AEW541 (▲) equivalent to its IC_20_. Data are presented as means ± standard deviation of a representative experiment (n = 3). **(B)** Molecular effects of NVP-AEW541 and lapatinib treatments. Cells were treated 24 h after seeding with a concentration equivalent to the IC_20_ of NVP-AEW541, lapatinib, or their combination. After 72 h, 50 ng/ml of IGF-I, EGF or both were added for 20 min, and expression of IGF-IR and EGFR pathway components was analyzed by Western blot. **(C)** Schematic representation of the molecular mechanism involved in NVP-AEW541 and lapatinib synergistic effect.

Resistance to individual treatment with the IGF-IR and EGFR/Her-2 inhibitors NVP-AEW541 and lapatinib, respectively, has been reported, reflecting the operation of compensatory mechanisms between the two pathways. To evaluate whether the individual effects of these drugs are potentiated by concurrent inhibition of both pathways, we assayed these two drugs in combination in the five cell lines. Increasing concentrations of lapatinib were combined with a fixed (IC_20_) concentration of NVP-AEW541. When used in combination, these drugs exhibited very potent synergy in all cell lines, with coefficients of drug interaction (CDIs) clearly < 0.7; remarkably, in some cases, CDI values were < 0.1 (Figure 2A).

To evaluate the effects of these drugs on the intracellular signaling activity of both pathways, we selected the NP-29 cell line, which exhibited the lowest CDI. In control cells, an IGF-I stimulus promoted substantial Akt and IRS-1 (Y612) phosphorylation and a small increase in IRS-1 (Y896) phosphorylation, but did not affect Erk1/2 phosphorylation. This suggests that the activity of the Ras-MAPK pathway is independent of IGF-I in these cells. Conversely, EGF stimulation resulted in elevated phosphorylation of Erk1/2, IRS-1 (Y612), and IRS-1 Y896 (Figure 2B). Inhibition of IGF-IR by NVP-AEW541 decreased IGF-I-induced phosphorylation of Akt and IRS-1 (Y612). In cells stimulated with EGF or IGF-I + EGF, NVP-AEW541 treatment increased EGFR pathway activation to a greater degree than in control cells, enhancing phosphorylation of Erk1/2, IRS1 (Y612), and IRS1 Y896 (Figure 2B). Whereas treatment with lapatinib diminished EGF-stimulated activation of Erk1/2 and IRS-1 (Y896), it did not significantly attenuate IGF-I- or IGF-I + EGF-induced activation of Akt and IRS-1 (Y612) (Figure 2B). Interestingly, simultaneous inhibition of both IGF-IR and EGFR/Her-2 by NVP-AEW541 and lapatinib completely abrogated IGF-I-, EGF-, and IGF-I + EGF-stimulated phosphorylation of Akt, Erk1/2, IRS-1 (Y612) and IRS-1 (Y896), confirming at the molecular level the strong synergy observed in cytotoxicity experiments (Figure 2B,C).

### Effect of IGF-IR and/or EGFR/Her-2 inhibition on tumorspheres viability

The role of CSCs in the resistance to different drugs has been extensively reported in recent years. Thus, the potent synergy obtained in tumor cells prompted us to examine the effects of NVP-AEW541 and lapatinib on cell viability in tumorspheres. These experiments were performed using the two cell lines that exhibited the highest synergy and in BxPC3 cells, a commercially available cell line previously reported to be capable of forming tumorspheres [35,36] that also exhibited a potent synergy (Additional file 1: Figure S1). An analysis of morphology and cell cycle profile in tumorspheres obtained from CP15T and BxPC3 cells revealed PCSC characteristics, but PCSC enrichment in NP-29 cells was questionable (Additional file 2: Figure S2A,B, Additional file 3: Supplemental methods).

**Figure 3:**
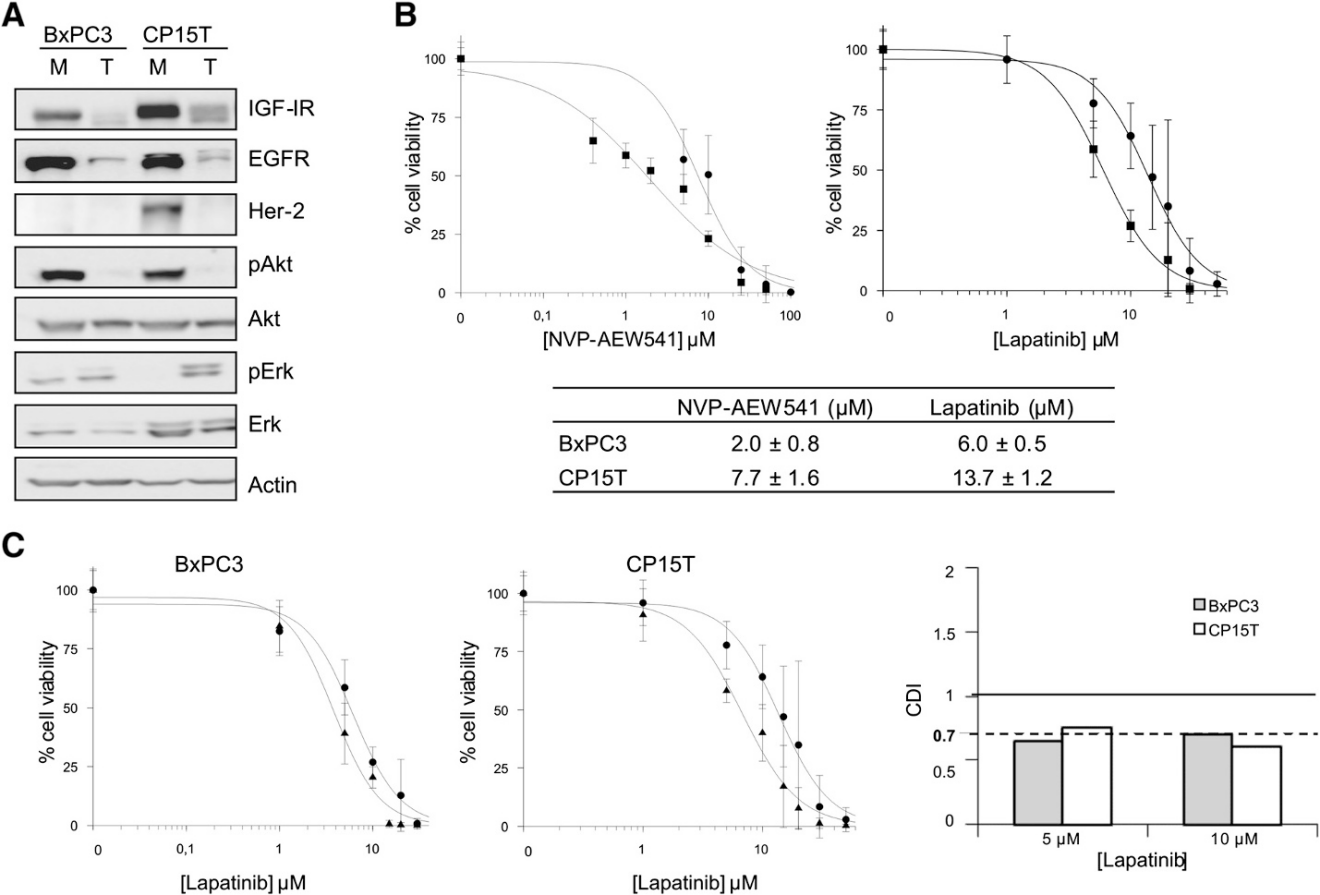
**IGF-IR and EGFR/Her-2 inhibition decreases the viability of pancreatic tumorspheres. (A)** Basal levels of IGF-I and ErbB receptors and their signaling pathway components in BxPC3 and CP15T tumorspheres were determined by Western blot. M, monolayer; T, tumorspheres. **(B)** Dose–response curves and IC_50_ values for NVP-AEW541 and lapatinib. Cells were seeded with increasing concentrations of NVP-AEW541 or lapatinib, and cell viability was measured by WST-8 assay 72 h after initiating treatment. ● BxPC3, ■ CP15T **(C)** Dose–response curves and CDI values for NVP-AEW541 and lapatinib combinations. Cells were seeded with increasing concentrations of lapatinib alone (●) or combined with a fixed concentration of NVP-AEW541 (▲) equivalent to its IC_20_. Data are presented as means ± standard deviation of three experiments.

Expression levels of receptors and the activity of their pathways were then determined. These analyses showed significant decreases in receptor expression and Akt phosphorylation in the PCSC population (Figure 3A). Despite this, both inhibitors were able to kill 100% of cells, showing IC_50_ values in the same range as were obtained with the corresponding monolayers (Figure 3B, Additional file 1: Figure S1A). These results contrast with the resistance observed with gemcitabine (Additional file 2: Figure S2C). Interestingly, combining these two drugs improved their inhibitory effect on cell viability, yielding CDI values near 0.7, indicative of a potent synergistic effect, at all concentrations (Figure 3C).

## Discussion

Despite rapid advances on many fronts, PDAC remains one of the most difficult human malignancies to treat. The clinical outcome of patients with this disease has not improved since the approval of gemcitabine, indicating the need for novel therapeutic strategies based on a better understanding of the molecular basis of this disease [1]. In this context, several drugs designed to inhibit IGF-IR have been developed, reflecting the fact that this receptor is frequently overexpressed in PDAC and is associated with tumor progression and poor prognosis [13,17,19,37]. However, several clinical trials of IGF-IR inhibitors have failed, probably in part because of the activation of compensatory pathways [23,24]. ErbB receptors have been proposed as one mechanism involved in the resistance to these inhibitors [28,38]. Therefore, combined treatment with inhibitors of both IGF-IR and ErbB receptors would appear to be a good strategy for overcoming the emergence of resistance.

Inhibition of IGF-IR and EGFR/Her-2 by NVP-AEW541 and lapatinib caused a concentration-dependent reduction of cell viability in all cell lines assayed. This cytotoxic effect has been previously described in other models, and, interestingly, it is tumor-selective, as it is higher in tumoral cells than in normal cells [39,40]. An evaluation of the basal expression of IGF-IR and ErbB family receptors and signaling pathway proteins showed no correlation between the levels of these receptors and sensitivity to their inhibition, in good agreement with previous results in several types of cancer [38,41,42]. Moreover, when used in combination, NVP-AEW541 and lapatinib strongly synergized in all cell lines at all concentrations assayed. Using other inhibitors, this potentiation has been reported in PDAC [38] and other tumors [43-45].

An analysis of the changes in signaling produced by single and combined treatments argue in favor of cross-talk between IGF-IR and ErbBs pathways upstream of their confluence at the MAPK and Akt level. IRS-1 is generally considered to be a unique substrate of IGF-IR, which phosphorylates IRS-1 at Y612. Notwithstanding this presumption, a more recent study on breast cancer suggests that EGFR has the ability to recruit and phosphorylate IRS-1 at Y896 [46]. This competence for the same substrate is supported by our results and could contribute to the resistance caused by activation of mutual compensatory pathways. The IRS-1 phosphorylation pattern clearly indicated that blocking IGF-IR signaling strongly induced phosphorylation of IRS-1 at Y896. This increase in IRS-1 phosphorylation highlights the crucial influence of this new mechanism—activation of MAPK and especially Akt phosphorylation—in the resistance to IGF-IR inhibitors, and points to preferential channeling of ErbB receptor signaling to IRS-I (Y896) phosphorylation via phosphorylated Akt. Interestingly, when both receptors were inhibited, IRS-1, Akt and MAPK phosphorylation were completely abolished, reinforcing the utility of combined inhibition of both pathways in averting the resistance induced by individual treatments.

Despite these good *in vitro* results, the outcome in patients has been disappointing. One possible reason for the failure of these targeted drugs could be the role of PCSCs in resistance [47,48]. The importance of the IGF-IR pathway in treatments targeting PCSCs has not been previously described, although several recent reports have demonstrated an association of this receptor with cell stemness in some tumors [49,50]. Our results showed that pancreatic cancer tumorspheres were sensitive to treatment with either NVP-AEW541 or lapatinib, in contrast to their high resistance to gemcitabine. Remarkably, combining both drugs again produced a synergistic effect similar to that observed in monolayers. This synergy in tumorspheres, which has not been previously described, indicates that inhibition of both pathways in PCSCs can also overcome the resistance caused by these compensatory pathways in this subpopulation.

## Conclusions

Simultaneous inhibition of IGF-IR and ErbB receptors by NVP-AEW541 and lapatinib circumvented the resistance observed at the molecular level with individual treatments. Interestingly, these inhibitors were also able to eliminate PCSCs, overcoming their resistance to conventional chemotherapy. Thus, the synergy observed with this combined treatment indicates that it may be possible to maximize patient benefit with the appropriate combination of currently known anticancer agents.

## Additional files

Additional file 1: Figure S1. Effect of NVP-AEW541 and lapatinib in the BxPC3 monolayers. (A) Dose–response curves and IC_50_ values for NVP-AEW541 and lapatinib. Cells were seeded with increasing concentrations of NVP-AEW541 or lapatinib, and cell viability was measured by WST-8 assay 72 h after starting treatment. Data are presented as means ± standard deviation of three experiments. (B) Dose–response curve and CDI values for NVP-AEW541 and lapatinib combination. Twenty-four hours after seeding, cells were treated with increasing concentrations of lapatinib alone (●) or combined with a fixed concentration of NVP-AEW541 (▲) equivalent to its IC_20_. Data are presented as means ± standard deviation of three experiments.
Additional file 2: Figure S2. Characterization of tumorspheres obtained from different human pancreatic cancer cell lines. (A) Morphology of BxPC3, CP15T, and NP-29 tumorspheres. Cells were maintained under standard culture conditions (monolayers) or in stem cell medium on ultra-low-adhesion plates (tumorspheres). Scale bar = 5 μm. (B) Cell cycle profiles of monolayers and tumorspheres. S-phase represented in light grey, G2/M-phase in dark grey, and G0/G1-phase in black. (C) Dose–response curve and IC_50_ values of gemcitabine for monolayers and tumorspheres. Cells were seeded with increasing concentrations of gemcitabine, and cell viability was measured by WST-8 assay 72 h after starting treatment. Data are presented as means ± standard deviation of three experiments. ■BxPC3 monolayer, □BxPC3 tumorspheres, ●CP15T monolayer, ○CP15T tumorspheres.
Additional file 3:
**Analysis of cell cycle by flow cytometry.**

## Abbreviations

CDI: Coefficient of drug interaction
CSC: Cancer stem cells
EGF: Epidermal growth factor
EGFR: Epidermal growth factor receptor
Erk: Extracellular signal-regulated kinase
IC_50_: 50% inhibitory concentration
IGF: Insulin-like growth factor
IGF-IR: Insulin-like growth factor-1 receptor
IRS-1: Insulin receptor substrate 1
MAPKs: Mitogen-activated protein kinases
pAkt: Phosphorylated Akt
PCSC: Pancreatic cancer stem cells
PDAC: Pancreatic ductal adenocarcinoma
