# Supplementary material for: Human pancreatic cancer stem cells are sensitive to dual inhibition of IGF-IR and ErbB receptors
Source: BMC Cancer. 2015 Apr 4;15:223. doi: 10.1186/s12885-015-1249-2 (PMC4403908; doi:10.1186/s12885-015-1249-2)
Supplement: Additional file 1: Figure S1. — Effect of NVP-AEW541 and lapatinib in the BxPC3 monolayers. (A) Dose–response curves and IC50 values for NVP-AEW541 and lapatinib. Cells were seeded with increasing concentrations of NVP-AEW541 or lapatinib, and cell viability was measured by WST-8 assay 72 h after starting treatment. Data are presented as means ± standard deviation of three experiments. (B) Dose–response curve and CDI values for NVP-AEW541 and lapatinib combination. Twenty-four hours after seeding, cells were treated with increasing concentrations of lapatinib alone (●) or combined with a fixed concentration of NVP-AEW541 (▲) equivalent to its IC20. Data are presented as means ± standard deviation of three experiments. [file 12885_2015_1249_MOESM1_ESM.pdf]

A

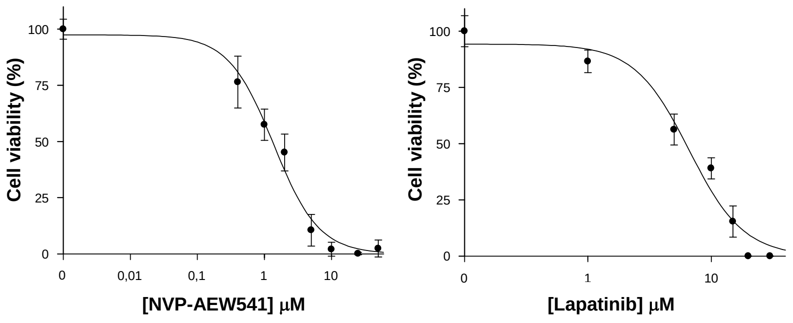

	NVP-AEW541 (μM)	Lapatinib (μM)
BxPC3	1.4 ± 0.2	6.6 ± 1.1

B

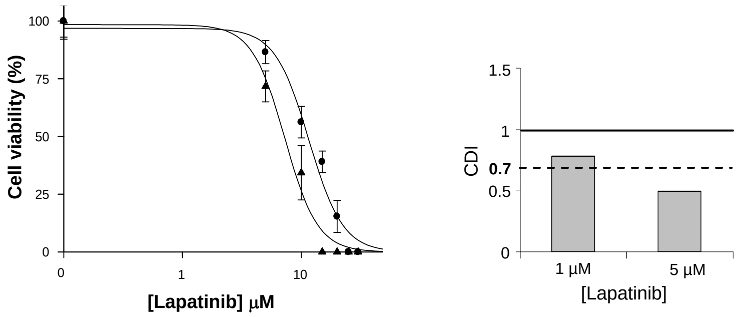

Effect of NVP-AEW541 and lapatinib in the BxPC3 monolayers. (A) Dose-response curves and IC50 values for NVP-AEW541 and lapatinib. Cells were seeded with increasing concentrations of NVP-AEW541 or lapatinib, and cell viability was measured by WST-8 assay 72 h after starting treatment. Data are presented as means ± standard deviation of three experiments. (B) Dose-response curve and CDI values for NVP-AEW541 and lapatinib combination. Twenty-four hours after seeding, cells were treated with increasing concentrations of lapatinib alone (●) or combined with a fixed concentration of NVP-AEW541 (▲) equivalent to its IC20. Data are presented as means ± standard deviation of three experiments.
