# Supplementary material for: Human pancreatic cancer stem cells are sensitive to dual inhibition of IGF-IR and ErbB receptors
Source: BMC Cancer. 2015 Apr 4;15:223. doi: 10.1186/s12885-015-1249-2 (PMC4403908; doi:10.1186/s12885-015-1249-2)
Supplement: Additional file 2: Figure S2. — Characterization of tumorspheres obtained from different human pancreatic cancer cell lines. (A) Morphology of BxPC3, CP15T, and NP-29 tumorspheres. Cells were maintained under standard culture conditions (monolayers) or in stem cell medium on ultra-low-adhesion plates (tumorspheres). Scale bar = 5 μm. (B) Cell cycle profiles of monolayers and tumorspheres. S-phase represented in light grey, G2/M-phase in dark grey, and G0/G1-phase in black. (C) Dose–response curve and IC50 values of gemcitabine for monolayers and tumorspheres. Cells were seeded with increasing concentrations of gemcitabine, and cell viability was measured by WST-8 assay 72 h after starting treatment. Data are presented as means ± standard deviation of three experiments. ■BxPC3 monolayer, □BxPC3 tumorspheres, ●CP15T monolayer, ○CP15T tumorspheres. [file 12885_2015_1249_MOESM2_ESM.pdf]

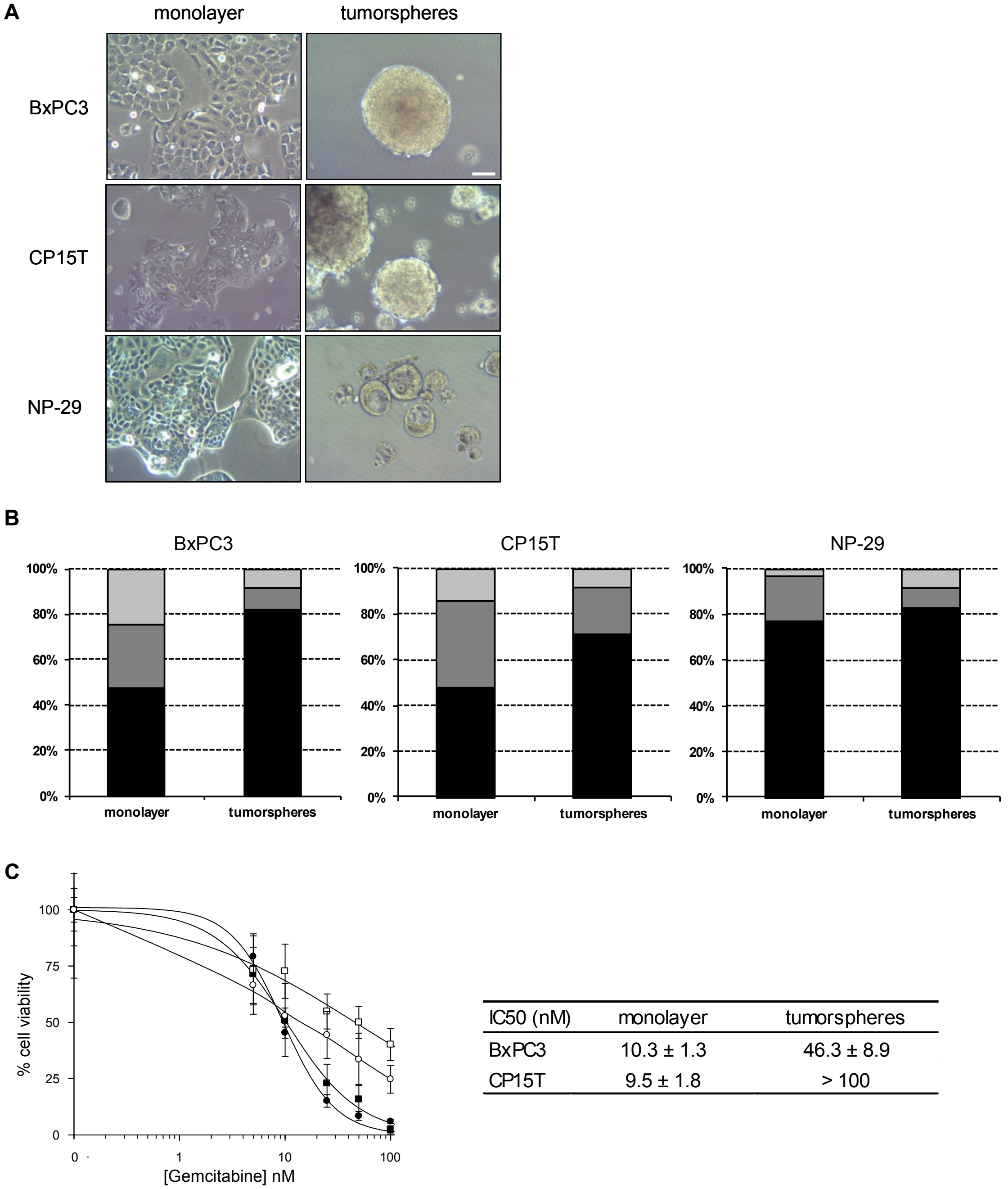

IC50 (nM)	monolayer	tumorspheres
BxPC3	10.3 ± 1.3	46.3 ± 8.9
CP15T	9.5 ± 1.8	> 100

Characterization of tumorspheres obtained from different human pancreatic cancer cell lines. (A) Morphology of BxPC3, CP15T, and NP-29 tumorspheres. Cells were maintained under standard culture conditions (monolayers) or in stem cell medium on ultra-low-adhesion plates (tumorspheres). Scale bar = 5 µm. (B) Cell cycle profiles of monolayers and tumorspheres. S-phase represented in light grey, G_2/M-phase in dark grey, and G_0/G_1-phase in black. (C) Dose-response curve and IC_{50} values of gemcitabine for monolayers and tumorspheres. Cells were seeded with increasing concentrations of gemcitabine, and cell viability was measured by WST-8 assay 72 h after starting treatment. Data are presented as means ± standard deviation of three experiments. ■ BxPC3 monolayer, □ BxPC3 tumorspheres, ● CP15T monolayer, ○ CP15T tumorspheres.
