# Supplementary material for: Human pancreatic cancer stem cells are sensitive to dual inhibition of IGF-IR and ErbB receptors
Source: BMC Cancer. 2015 Apr 4;15:223. doi: 10.1186/s12885-015-1249-2 (PMC4403908; doi:10.1186/s12885-015-1249-2)
Supplement: Additional file 3: — Analysis of cell cycle by flow cytometry. [file 12885_2015_1249_MOESM3_ESM.pdf]

Analysis of cell cycle by flow cytometry

Cells were harvested and fixed with 70% ethanol (v/v). After collecting by centrifugation at 4 000 rpm for 4 min, cells were stained by incubating for 1 h at 4ºC with a solution of 100 µg/ml propidium iodide in phosphate-buffered saline containing 10 µg/ml RNase A. The cell cycle distribution of propidium iodide-stained cells was analyzed using a Lab Cell Quanta flow cytometer (Beckman Coulter Inc., Miami, FL, USA).
